# Supplementary figures and images for: PI3Kdelta-driven expansion of regulatory B cells impairs protective immune responses to Trypanosoma congolense parasite infection
Source: PLoS Pathog. 2025 Nov 5;21(11):e1013663. doi: 10.1371/journal.ppat.1013663 (PMC12604799; doi:10.1371/journal.ppat.1013663)

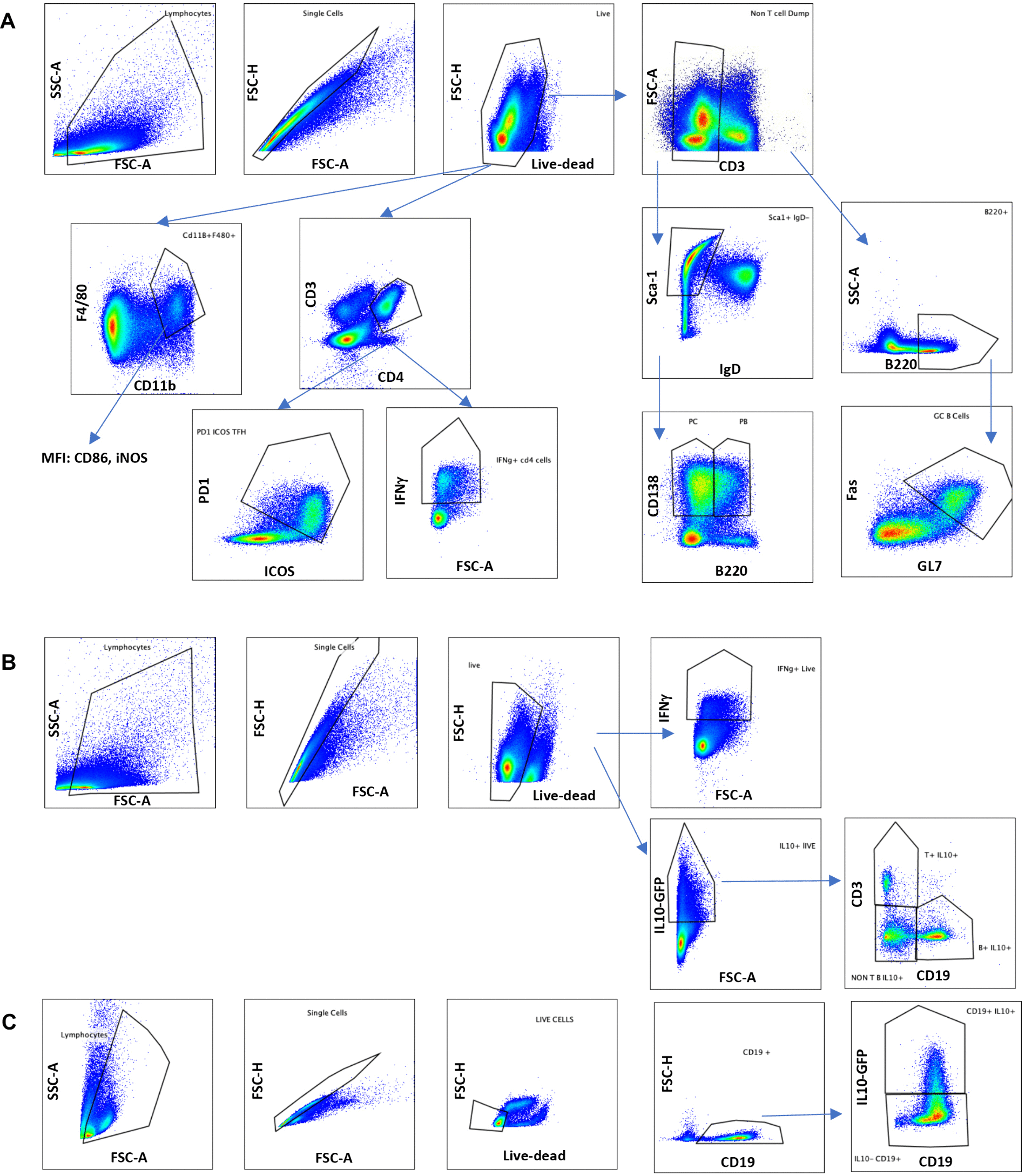

Supplement: S1 Fig — (A) Spleen B, T & Macrophage cell populations, (B) Spleen total frequencies of IFNγ-producing & IL10-producing cells. Bottom right shows subsetting of IL10+ cells to calculate absolute numbers of IL10-producing B, T and non-B/T cells. (C) Peritoneal IL10-producing B cells. (TIF) [file ppat.1013663.s001.tif]

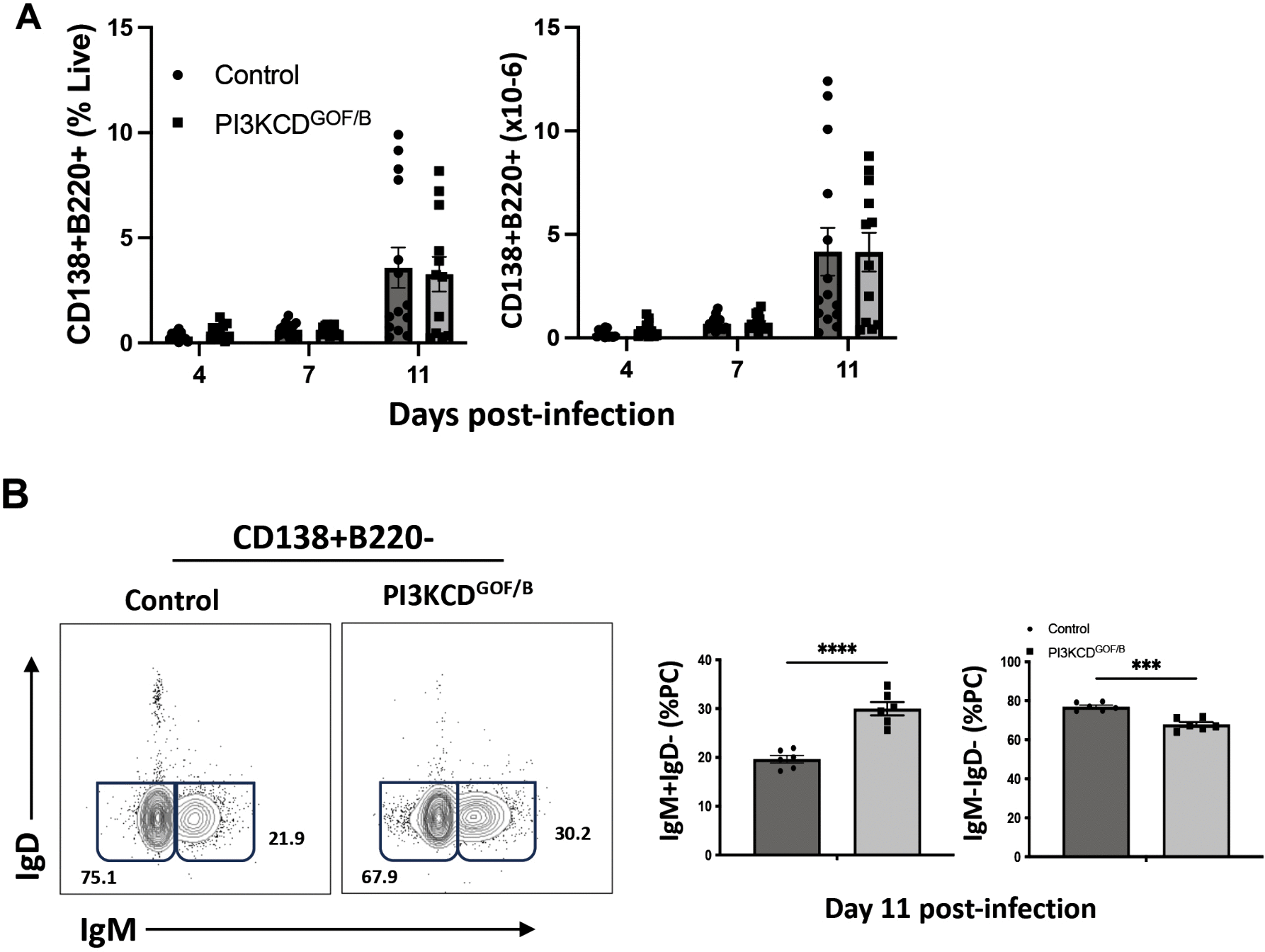

Supplement: S2 Fig — Frequencies of (A) Plasmablasts and (B) IgM+ versus IgM- plasma cells in the spleen at the indicated days post-infection. (TIF) [file ppat.1013663.s002.tif]

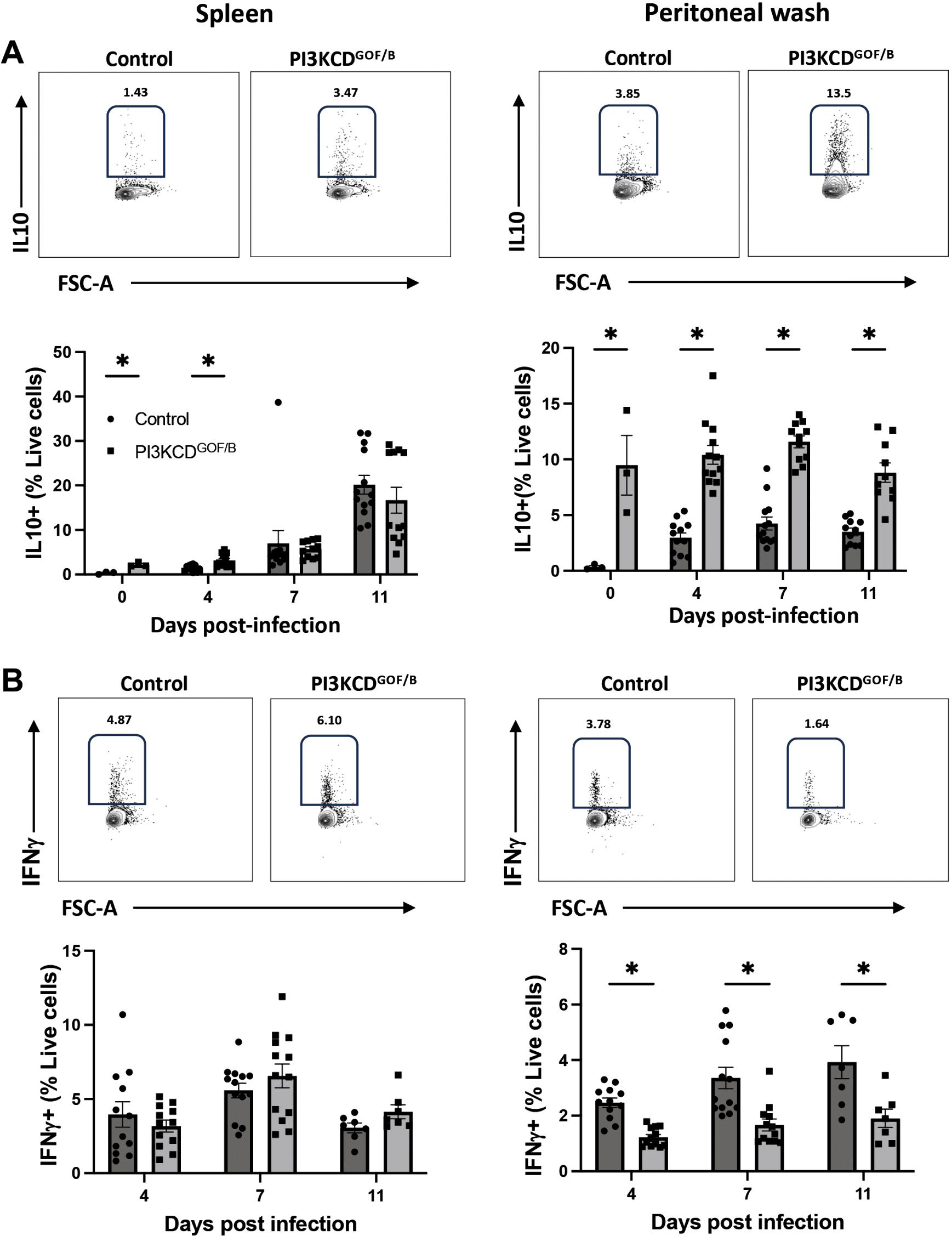

Supplement: S3 Fig — Frequencies of (A) IL10+ or (B) IFNγ+ cells within spleen (left) or peritoneal cavity (right) at the indicated days post-infection. (TIF) [file ppat.1013663.s003.tif]

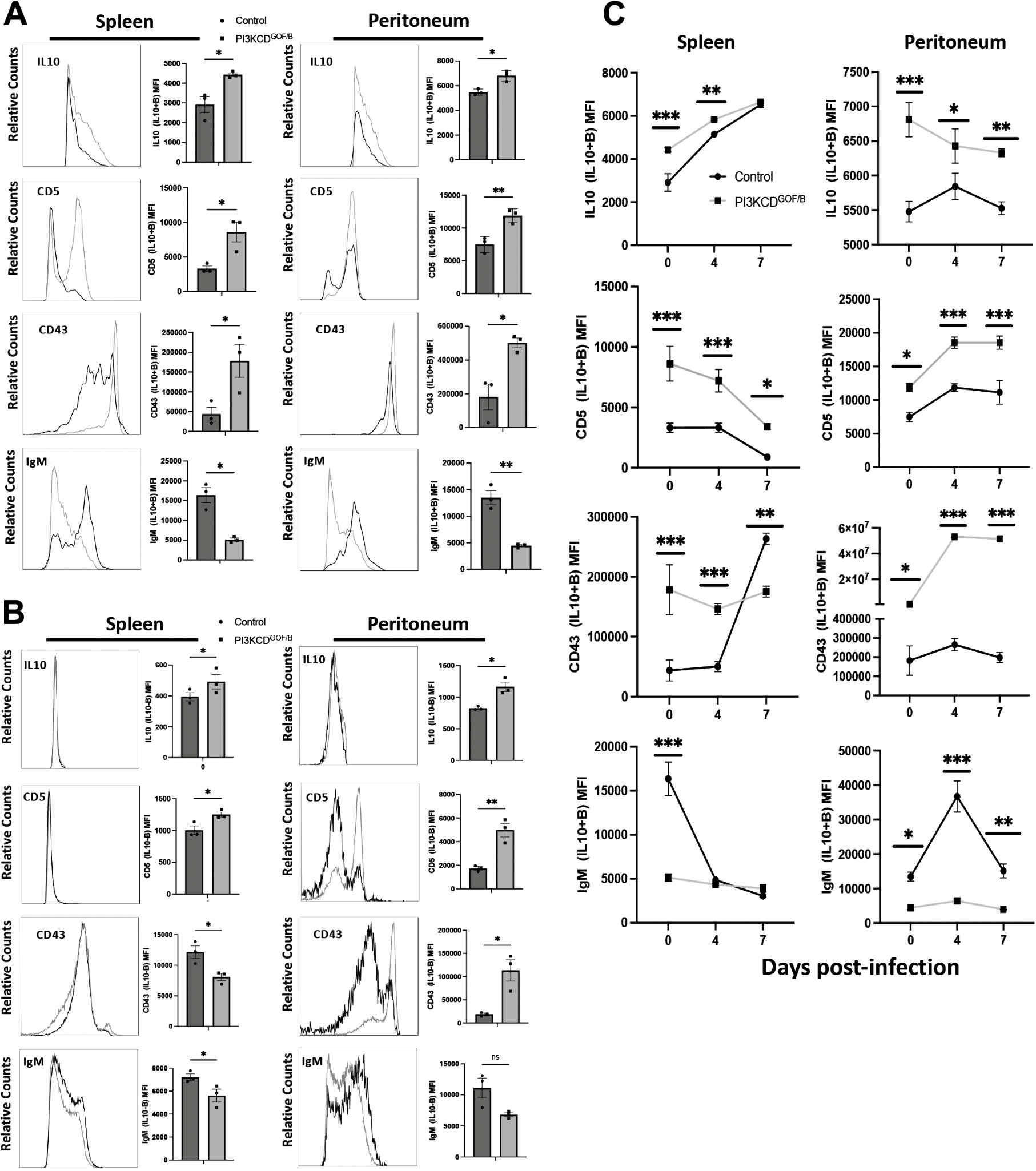

Supplement: S4 Fig — Phenotype of (A) IL10-positive B cells at baseline (gated live CD19+IL10GFP+ cells), (B) IL10-negative B cells at baseline (gated live CD19+IL10GFP- cells), and (C) IL10-positive B cells at infection (gated live CD19+IL10GFP+ cells), from control or PI3KDGOF/B spleen or peritoneal cavity were analyzed for levels of IL10GFP, CD5, CD43 or IgM expression. (TIF) [file ppat.1013663.s004.tif]

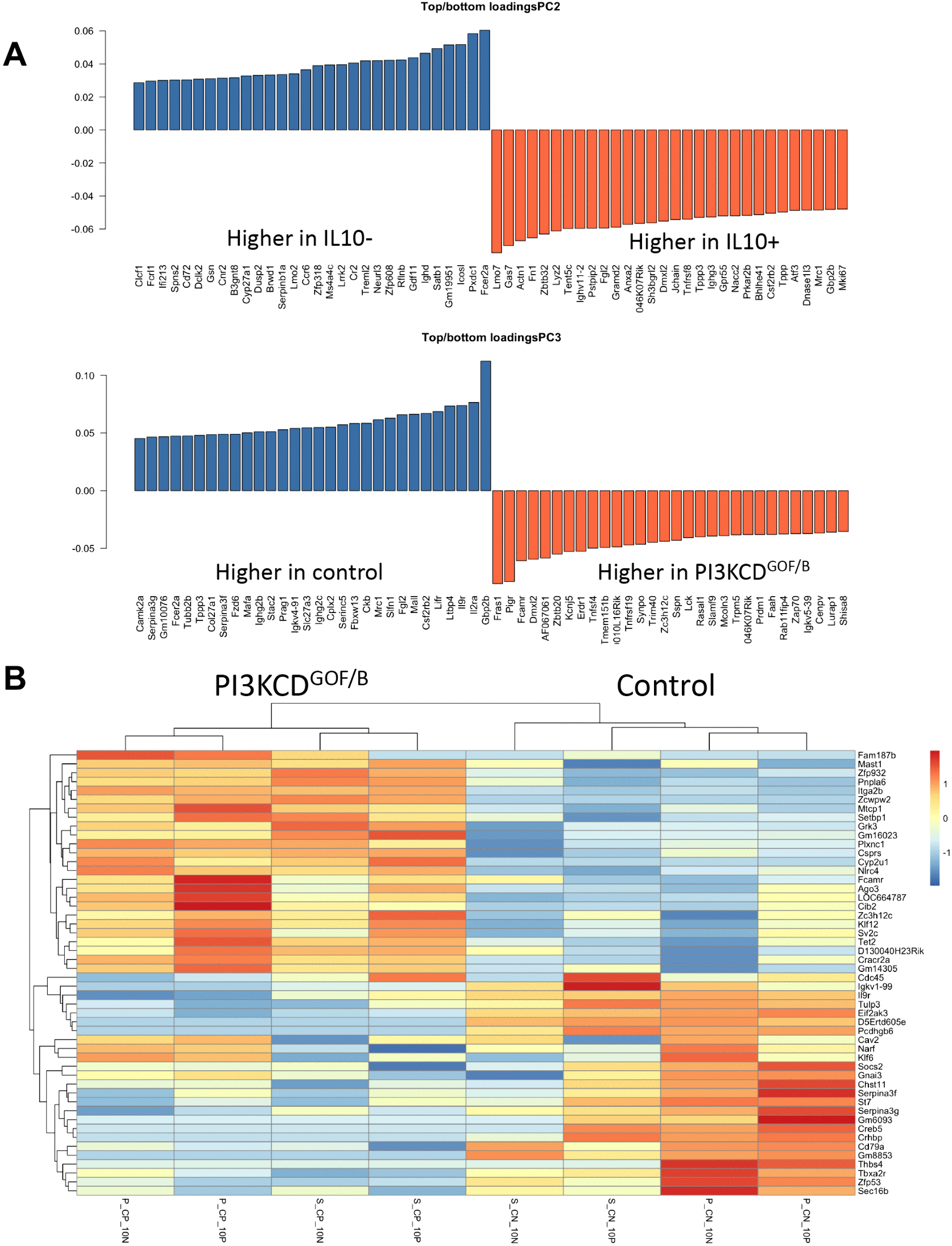

Supplement: S5 Fig — (A) Top genes driving variance for PC2 and PC3. (B) Heatmap depicting the top 50 differentially expressed genes according to adjusted p value, comparing PI3KCDGOF/B versus control groups. (TIF) [file ppat.1013663.s005.tif]

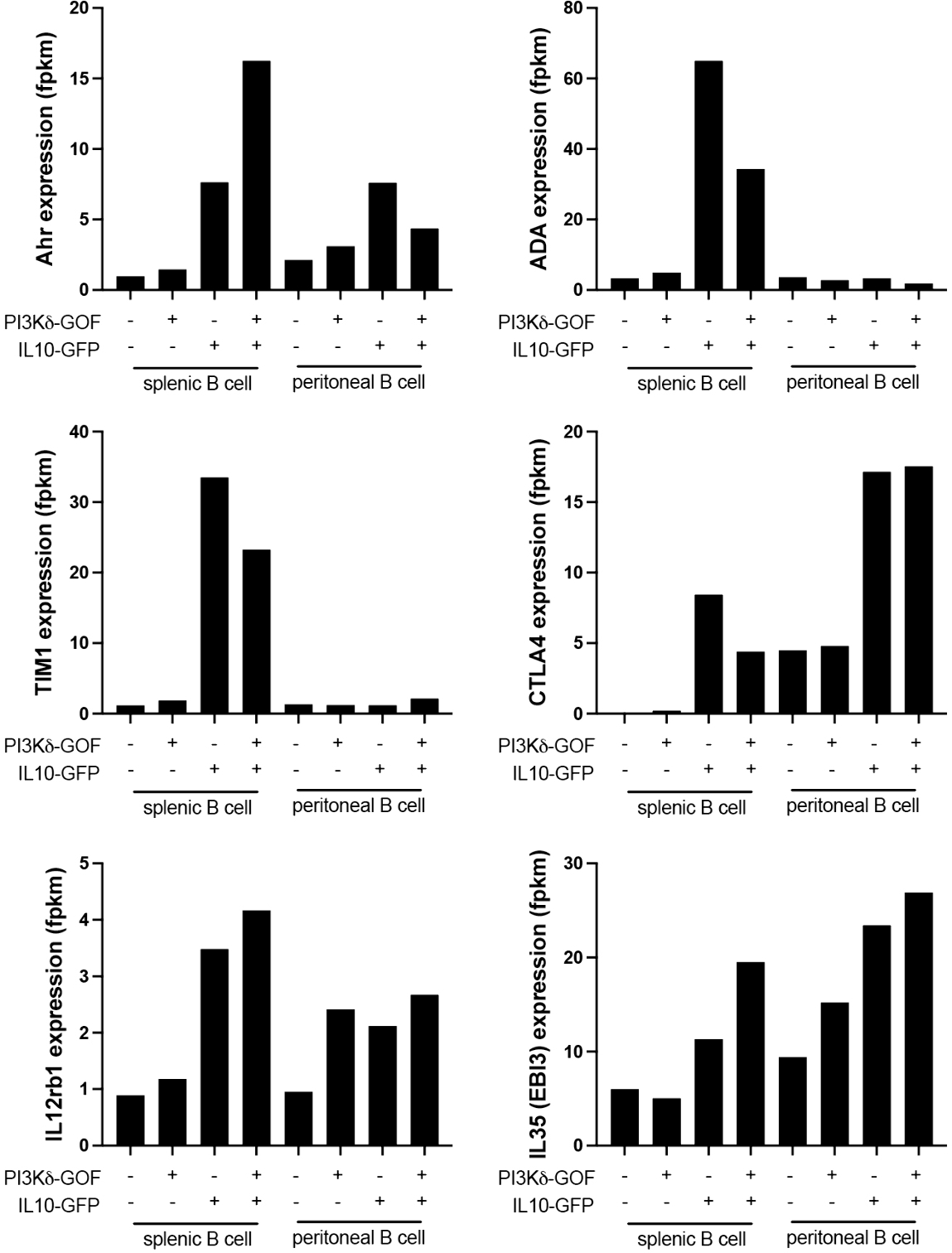

Supplement: S6 Fig — Determined from RNAseq data. (TIF) [file ppat.1013663.s006.tif]

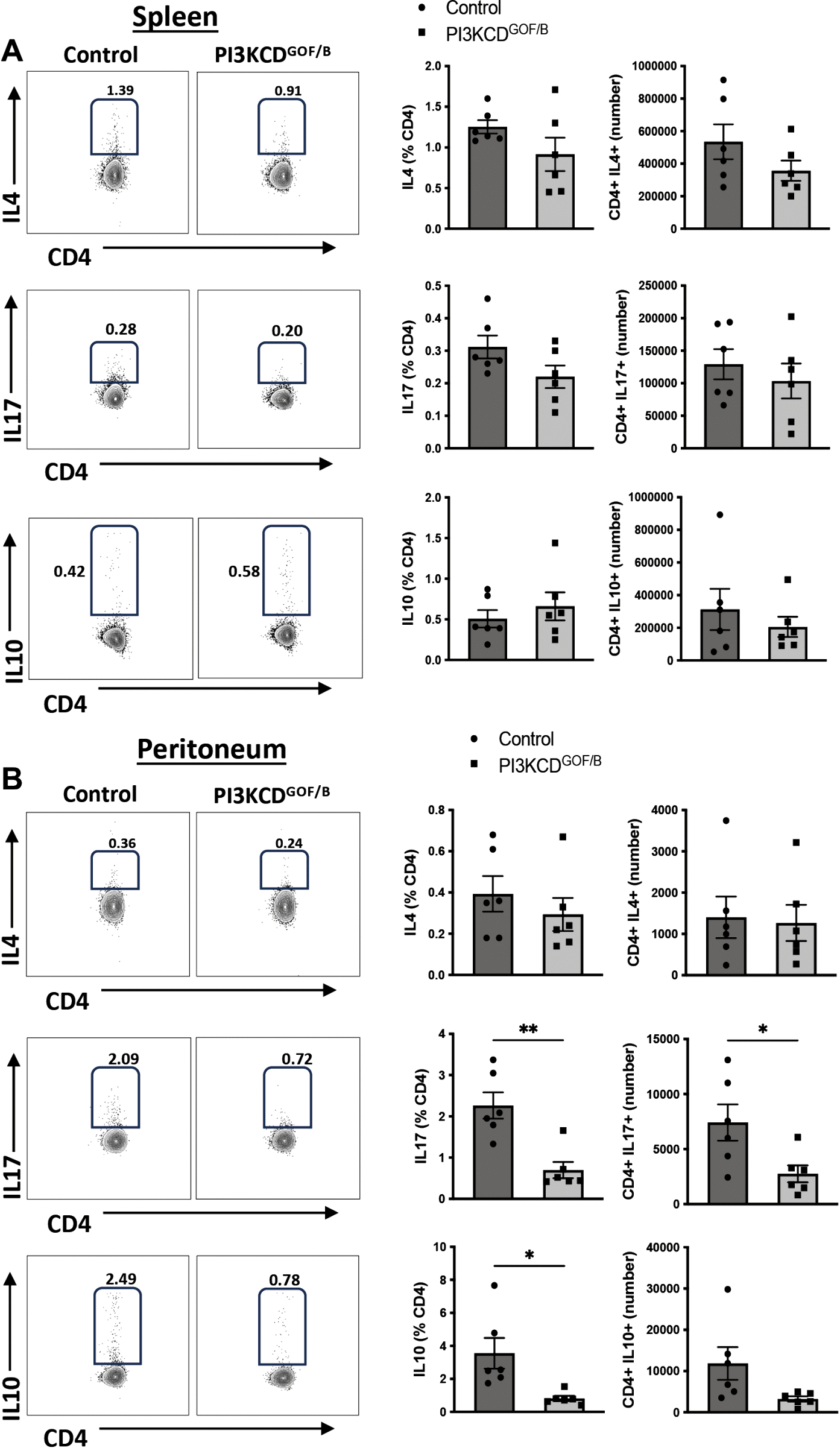

Supplement: S7 Fig — Frequencies of (A) splenic and (B) peritoneal IL4+, IL17+ or IL10+ CD4+ T cells were assessed and expressed as percentages and absolute numbers at the indicated day post-infection. (TIF) [file ppat.1013663.s007.tif]

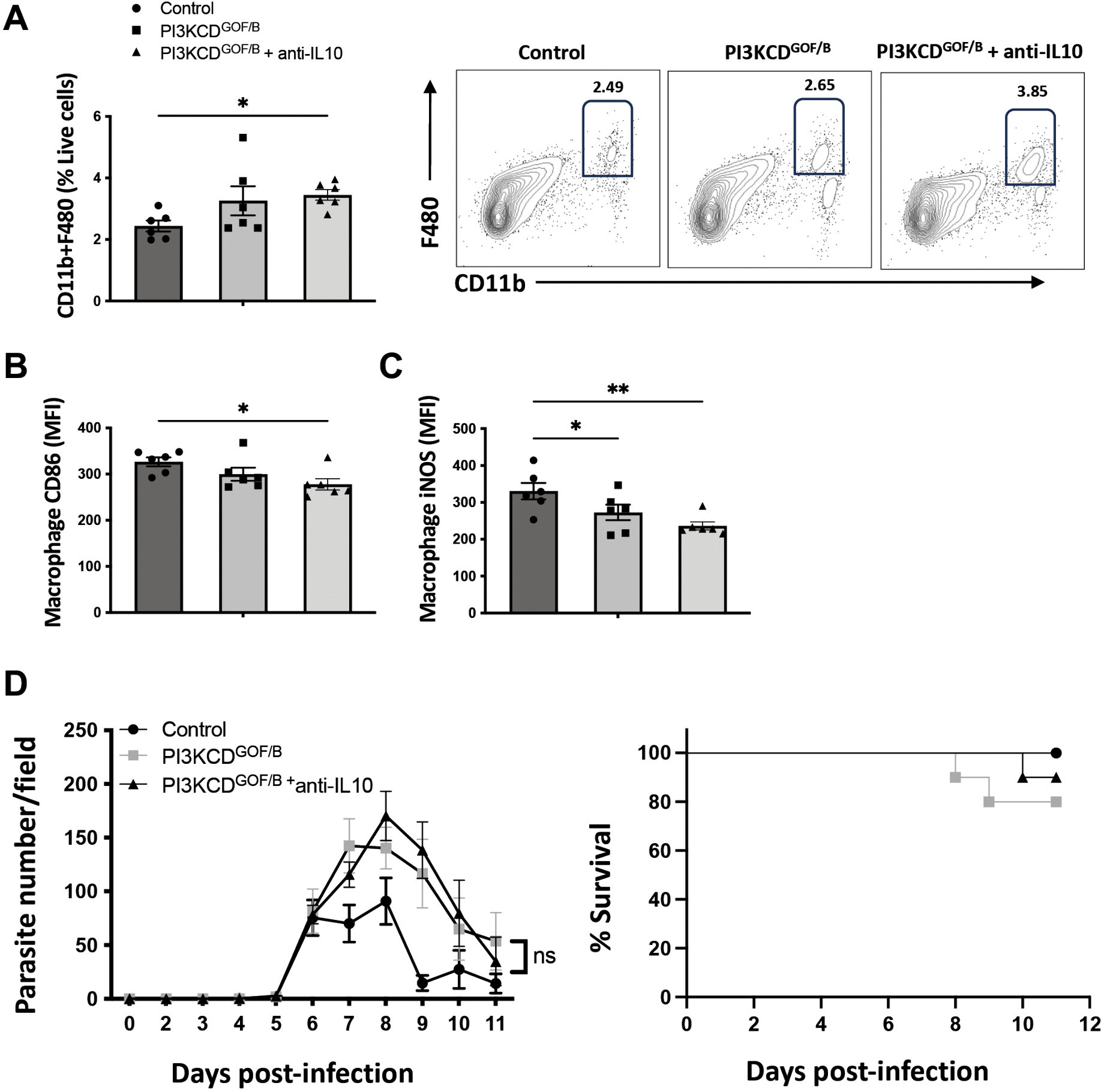

Supplement: S8 Fig — Splenic (A) Macrophage frequencies by flow cytometry, (B) CD86 expression and (C) iNOS expression at day 11 post-infection. (D) Parasitemia and survival curves following IL10 blocking in PI3KDGOF/B mice. (TIF) [file ppat.1013663.s008.tif]
