# Supplementary material for: PI3Kdelta-driven expansion of regulatory B cells impairs protective immune responses to Trypanosoma congolense parasite infection
Source: PLoS Pathog. 2025 Nov 5;21(11):e1013663. doi: 10.1371/journal.ppat.1013663 (PMC12604799; doi:10.1371/journal.ppat.1013663)
Supplement: Attachment — Submitted filename: Response to reviewers.docx [file ppat.1013663.s011.docx]

**Response to Reviewers**

We thank the reviewers for their comments and suggestions to improve the manuscript. We have undertaken new experiments and revisions further describing how PI3Kdelta gain of function mutation impacts immune responses to Trypanosome infection *(3 new supplementary figures as well as an additional figure panel*) and have now determined the impact of IL10 blockade as requested (a new figure and a new supplementary figure). According to the reviewer’s suggestions we have also reformatted all figures to show individual data points, added example flow cytometry plots in the main figures, and substantially shortened the introduction. The reviewer’s comments and our responses are summarized below.

**Reviewer #1**

1. IL-10-producing Breg can either be beneficial or pathological, depending on the context. I think it is important to show the function of Breg (either by depleting Breg or IL-10) in the context of B cell-specific GOF mutant mice and T. congolense infection.

*>A limitation in the area of regulatory B cell biology is the current lack of good tools to specifically deplete these cells without broadly impacting humoral immune responses, thus blockade of IL10 as suggested by the reviewer is often used to neutralize one of the regulatory molecules made by these cells. We have now performed IL10 blockade experiments and included the data in the manuscript (new Figure 9 and supplementary Figure 8). The results show that IL10 blockade did impact some aspects of the immune response including B cell, T cell and macrophage responses but this was not sufficient to normalize parasitemia. This suggests that expanded IL10-producing B cells likely utilize additional immunosuppressive mechanisms beyond IL-10 itself to regulate immune responses to Trypanosome infection. This is consistent with our results showing that IL10-producing B cells express multiple regulatory factors (Supplementary Figure 6), and consistent with other studies showing blockade of a single factor is not sufficient to neutralize Breg functional activity (discussion added in page 18). For example, Stark and colleagues demonstrated in a similar PI3Kdelta gain-of-function model that IL-10 blockade was insufficient to reduce disease susceptibility in Streptococcus pneumoniae lung infection (PMCID:* [*PMC6085315*](https://pmc.ncbi.nlm.nih.gov/articles/PMC6085315/)*). Notably, we and others have previously observed disease resistance in mice entirely lacking functional innate-like IL10-producing B cells, such as the PI3Kdelta-loss of function mice ( PMCID: PMC34380646, PMC27999013).*

2. To explain the generation of altered antibody isotypes, I think it is important to look at the proportion and number of cells expressing IgM and IgG by surface staining of GC B cells and intracellular staining for plasma blast/plasma cells.

*>As B cells can switch to multiple IgG isotypes it is not straightforward to quantify each of them by flow cytometry. However, we found that PI3KD GOF show a higher proportion of IgM+IgD- plasma cells and less IgM-IgD- isotype-switched plasma cells (Supplementary Figure 2B). We have highlighted in our discussions (page 17 ) the disconnect that can occur between total versus antigen-specific antibodies (much of the former presumably relates to the known polyclonal B cell activation during Trypanosome infection).*

3. Proportion IL10 producing B cells remain high at day 0 and have not increased during Trypanosome-infection (Figure 5). In fact, the proportion of IL10-producing B cells is decreased in the peritoneal cavity of GOF mice after infection. The number of IL-10-producing B cells remains high at day 4 post-infection. The author needs to show IL-10-producing B cell numbers at day 0 to support the claim that trypanosome infection generates higher IL-10-producing B cells.

*> Our results in Figure 5B strongly show elevated frequencies of IL10-producing B cells both at baseline and post-infection. To further highlight this, we have now added day 0 data to Figure 5C (absolute numbers of IL10-producing cells). The results show that in addition to elevated numbers of IL10-producing B cells at baseline, GOF mice show a greater increase in the number of these cells post-infection.*

**Reviewer #2**

1,The authors conclude that “that B cell-intrinsic PI3KCD hyperactivation can compromise macrophage activity and function”. Although the authors have previously characterized the B cell-intrinsic PI3KE1020K/b mice (Wray-Dutra et al), it would be useful to confirm that the mutant is not expressed in the macrophages after infection, as a control.

*> In two IL10 blockade experiments we sorted splenic B cells and macrophages from infected MB1-Cre+ and MB1-Cre- mice. Using a specific PCR assay to detect non-recombined and Cre-recombined alleles we were able to confirm the introduction of the mutation in B cells from Cre+ mice, but unfortunately, we obtained insufficient macrophage DNA to detect any specific bands. All mice used in this study are confirmed to be negative for germline Cre recombination using ear punch DNA, and there is no evidence in the literature that the widely-used B cell-specific Cre used here, (MB1-Cre) is expressed in macrophages.*

2. What happens to responses if mixed bone marrow chimeras are infected? Is this a dominant effect? Ultimately, is increased IL-10 responsible for these phenotypes? Could the authors use a titration of blocking anti-IL-10 to address this to see if there is a dose where death is reduced in the mutant mice? Some additional characterization would be helpful.

*>We have now performed IL10 blockade experiments and included the data in the manuscript (new Figure 9 and supplementary Figure 8). The results show that IL10 blockade did impact some aspects of the immune response including B cell, T cell and macrophage responses but this was not sufficient to normalize parasitemia. This suggests that expanded IL10-producing B cells likely utilize additional immunosuppressive mechanisms beyond IL-10 itself to regulate immune responses to Trypanosome infection. This is consistent with our results showing that IL10-producing B cells express multiple regulatory factors (Supplementary Figure 6), and consistent with other studies showing blockade of a single factor is not sufficient to neutralize Breg functional activity (discussion added in page 18). For example, Stark and colleagues demonstrated in a similar PI3Kdelta gain-of-function model that IL-10 blockade was insufficient to reduce disease susceptibility in Streptococcus pneumoniae lung infection (PMCID:* [*PMC6085315*](https://pmc.ncbi.nlm.nih.gov/articles/PMC6085315/)*). Notably, we and others have previously observed disease resistance in mice entirely lacking functional innate-like IL10-producing B cells, such as the PI3Kdelta-loss of function mice ( PMCID: PMC34380646, PMC27999013).*

3. It would also be useful to see what happens in mice that constitutively express the activated mutant in all cells (that express PI3Kd).

*>The purpose of our study is to examine how B cell-intrinsic dysregulation of PI3K impacts the immune response to Trypanosome infection. We have not conducted another study using germline mutants.*

**Reviewer #3: Major Comments:**
1. The authors report that PI3KCD hyperactivation diminishes antigen-specific IgG responses by day 11 while inducing a robust IgM response, both associated with higher parasite burdens. Although previous studies suggest PI3KCD hyperactivation impairs the development of antigen-specific antibodies, the role of these antibodies in controlling or sustaining parasite burden remains unclear. An adoptive transfer experiment using antibodies from infected WT and PI3KCDE1021K/B mice would help determine their role in modulating infection outcomes.

*>Our study primarily addresses the differential control of parasitemia at early time points prior to the generation of antigen-specific Antibodies. It would indeed be interesting to address other questions, such as whether subtle changes in antigen-specific antibody quality, quantity or kinetics in PI3KCDE1021K/B mutants may impact parasite control in the longer term. However, there is not a straightforward assay to examine this that can be performed during the revision period. The interpretation of antibody transfer experiments can be ambiguous and are not well suited to resolve subtle changes in antigen-specific antibody quality, quantity or kinetics - for example, we have previously shown that non-specific natural IgM antibody can play a significant role in early protection (PMID: 34380646).*

2. A notable expansion of IL-10-producing B cells is associated with increased parasite burden and mortality in PI3KCDE1021K/B mice; however, the direct role of IL-10 in this context is not fully elucidated. Blocking the IL-10 receptor in T. congolense-infected PI3KCDE1021K/B mice could provide mechanistic insights into IL-10’s role in immune regulation and its contribution to increased susceptibility associated with PI3KCD hyperactivation.

*>We have now performed IL10 blockade experiments and included the data in the manuscript (new Figure 9 and supplementary Figure 8). The results show that IL10 blockade did impact some aspects of the immune response including B cell, T cell and macrophage responses but this was not sufficient to normalize parasitemia. This suggests that expanded IL10-producing B cells likely utilize additional immunosuppressive mechanisms beyond IL-10 itself to regulate immune responses to Trypanosome infection. This is consistent with our results showing that IL10-producing B cells express multiple regulatory factors (Supplementary Figure 6), and consistent with other studies showing blockade of a single factor is not sufficient to neutralize Breg functional activity (discussion added in page 18). For example, Stark and colleagues demonstrated in a similar PI3Kdelta gain-of-function model that IL-10 blockade was insufficient to reduce disease susceptibility in Streptococcus pneumoniae lung infection (PMCID:* [*PMC6085315*](https://pmc.ncbi.nlm.nih.gov/articles/PMC6085315/)*). Notably, we and others have previously observed disease resistance in mice entirely lacking functional innate-like IL10-producing B cells, such as the PI3Kdelta-loss of function mice ( PMCID: PMC34380646, PMC27999013).*

3. The study shows that PI3KCD hyperactivation alters effector memory CD4 T cell differentiation, though no significant differences in IFNγ+ CD4 T cells were observed in infected PI3KCDE1021K/B mice. It would be valuable to also examine Th2, Treg, and Th17 populations, as these subsets contribute regulatory cytokines like IL-10 that might further elucidate the observed immune dysregulation.

*> As requested, we have examined the impact of B cell-intrinsic PI3KCD hyperactivation on other T cell subsets. On examination of CD4+ T cell production of IL4, IL17 and IL10 at day 11, we observed evidence of additional alterations in T cell functional responses, including a significant reduction of IL17+ cells in the peritoneal cavity. Results are presented in a new Supplementary Figure 7 and discussed on page 17 of the manuscript.*

**Part III – Minor Issues: Editorial and Data Presentation Modifications**
Please use this section for editorial suggestions as well as relatively minor modifications of existing data that would enhance clarity.

Reviewer #1: Figure quality is poor, which is very prominent in Figure 6. Figures 6 and 7 are hard to read.

*> This was caused by file conversion in the manuscript submission system and will be corrected on upload*

Reviewer #2: The authors show summary graphs for most of the data in the paper. It would be helpful if they showed individual data points. The authors show a representative gating strategy in Figure S1, but it would be useful to include the flow plots for the measured parameters in the main figures.

*> As requested we have changed graphs to show individual data points*

*> As requested we have moved some representative flow plots to Figures 3 and 8*

In Fig S4 it would be helpful to show levels of these markers compared to that seen in the IL10- cells (even for the IL-10 to show the negative levels).

> We have added the requested phenotype data for IL10-negative B cells to Figure Supplementary 4B

Reviewer #3: Minor Comments:
1. The introduction is overly detailed and could be condensed to 3-4 paragraphs, focusing on key background information relevant to the study’s primary findings.

> As requested we have edited and shortened the introduction by about half.

2. The manuscript would benefit from representative flow cytometry dot plots accompanying the quantification graphs, improving clarity and interpretation of the data.

*> As requested we have moved some representative flow plots to Figures 3 and 8*
