# Supplementary material for: PI3Kdelta-driven expansion of regulatory B cells impairs protective immune responses to Trypanosoma congolense parasite infection
Source: PLoS Pathog. 2025 Nov 5;21(11):e1013663. doi: 10.1371/journal.ppat.1013663 (PMC12604799; doi:10.1371/journal.ppat.1013663)
Supplement: Attachment — Submitted filename: Response to Reviewers (rereview) Oct 19.docx [file ppat.1013663.s012.docx]

**Response to Reviewers**

We thank the reviewers for their comments and suggestions to improve the manuscript. We have completed the additional revisions requested after the second round of review. Please find below our point-by-point response to reviewers.

**Additional Editor Comments:**

Comments from all reviewers for this revised manuscript are shown below for your information.

A. Please add day 0 data to Figure 4.

****Done. As expected, only baseline IL10 levels were found to be different in PI3KD-GOF/B mice*

B. and a more detailed flow cytometry characterization of IL10 producing populations in infected mice.

**** Done – new Supplementary Figure 4C. We found that within the IL10+ B cell population some markers changed over time after infection, likely reflecting both activation of pre-existing IL10+ cells and induction of new IL10+ B cell populations*

C. Please also add representative plots for all data before resubmitting

*** *Done. Representative flow plots added to Figures 5B, 9B/C, S3A/B, S7A/B, S8A*

Reviewer #2: 
I was not one of the initial reviewers of this paper, but have evaluated the responses to the first set of reviews. The effect of IL-10 blockade was most pronounced on antibody class-switching, with some effects on iNOS production by macrophages. The authors’ interpretation of the limitations of this approach is reasonable, as there may be other mechanisms by which Bregs influence the anti-parasite response

-the authors could strengthen their discussion by including other possibilities such as whether the altered Ig classes may contribute to the disease (and affect other immune parameters).

**** the potential impact of altered Ig subclasses is discussed in lines 416-428*

-In addition to the day 0 data which is now included in Figure 5C, data at day 0 for Figure 4 would strengthen the manuscript. This would be especially beneficial for Figures 4A, D, and E, since there are already differences in IL-10 and IFN-g at the d4 timepoint. I agree that the authors should include representative flow plots for all data (for example, new data).

****Day 0 data has now been added to Fig 4. Only baseline IL10 levels were found to be different in PI3KD-GOF/B mice (as expected based on increased frequencies of cells expressing the IL10 reporter).*

Additional comments include:
The authors suggest that altered cytokine with reduced IFNg:IL10 ratios contributes to the poor initial responses. However, in the IL-10 blocking experiments, they only look at day11, which is useful for the Ig class-switching and macrophage iNOS, but IFNg differs most at d4. Were there differences in cytokines earlier? Was TNF-a affected at d11 by blocking IL-10, as that was quite different at the later days in their original data?

*** *definitely day 4 would be the best timepoint for determining the effect of IL10 blockade on generation of cytokine responses, however given the lack of effect of IL10 blockade on controlling early parasitemia in PI3KD-GOF/B mice we have not prioritized more detailed analyses of IL10 blockade at earlier time points. As requested we examined TNFa at day 11 (figure inserted). At this time point the TNF levels appear similar between control and GOF mice and anti-IL10 treatment somewhat reduced TNFa levels in GOF mice. The interpretation at late time points is complex, but this might relate to slightly improved control of infection with anti-IL10 due to improved antibody response and elevated iNOS in macrophages - leading to faster decline of TNF levels.*

**TNFalpha (pg/ml)**

**TNFalpha (pg/ml)**

**serum**

**peritoneal wash**

If this was the initial review, additional comments include:
1. Given the hyper-IgM/reduced class switch antibody phenotype in the mutant mice, it is possible that qualitative differences in the early antibody response could contribute to the susceptibility to T. congolense. To address this possibility, the authors could cross the B cell-specific mutants to a strain incapable of producing soluble Ig or to a strain with an irrelevant BCR (the latter cross is relatively fast). This would remove soluble antibody specificity as a contributing factor to their observed phenotypes. However, as a re-review, this is not required.
2. The authors point out that the mutant mice have elevated IL-10 both at baseline and early during infection. Are differences in baseline IL-10 (or other factors) sufficient to impair T. congolense control, or does the Breg response to infection dictate susceptibility? Could this be addressed by depleting B cells at the time of infection? Alternatively, IL-10 blockade could be initiated at different timepoints shortly after infection, though this would not account for other effector molecules produced by Bregs. Again, I would have liked this if this was an initial review but would not require it for a re-review.

****these are good suggestions for follow-up studies. The question about the quality of the antibody response is an interesting one and we have discussed that in the manuscript. The difficulty with experiments removing soluble Ig entirely is that antibody is essential for surviving this infection. Acutely removing B cells entirely would be interesting but will not address the specific B cell-associated regulatory mechanisms. Our RNAseq and flow phenotyping data provide a number of other candidate regulatory molecules but extensive additional work will be required to identify mechanism(s) beyond IL10.*

Reviewer #4: As a round 1 revised paper some important questions were answered but, however, I have some concerns.

1) The authors show that PI3Kδ hyperactivation in B cells is not associated with impaired germinal center or plasma cell responses, based on the cell surface markers analysis by flow cytometry. However, I believe that a histological analysis of germinal centers (at least of the spleen) is necessary, as extrafollicular structures have been described in similar parasites infections.

****previous studies have shown that Trypanosoma infection causes polyclonal B cell activation and disruption of splenic architecture, making it difficult to histologically distinguish classical GC versus extrafollicular responses. It can be considered a “mixed” response in this regard, and the conclusion from flow cytometry data is simply that the frequencies of phenotypic B cell subsets generated are not obviously different in PI3KD-GOF/B mutant mice. We have added additional discussion addressing this (lines 405-410).*

2) The characterization of different IL-10-producing cell populations was performed in uninfected mice. However, a more detailed characterization of these cells in infected mice using flow cytometry is still required.

****We thank the reviewer for this suggestion. When we analyzed the phenotype of IL10+ B cells after infection we found that in some markers changed over time after infection and some differences between spleen and peritoneal cavity were noted. This likely reflects both activation of pre-existing IL10+ cells and induction of new IL10+ B cell populations.*

*We have added a new figure presenting this analysis (Supplementary Fig 4C) and some additional discussion on this point Lines 440-443.*

I realize that the introduction and discussion should be concise but the relevance of B cell and specially the Breg in other parasitic infection should be discussed.

****we have added in some additional discussion on Breg in other parasitic infections (lines 75-81)*

How were identified de IL-10 secreting cell by flow cytometry in Figure S4? If they were identified by intracytoplasmic immunofluorescence, describe in Materials and Methods.

****in all figures, the genetically encoded fluorescent reporter (known as IL10-GFP or VertX) was used to identify IL10-expressing cells. The details of the reporter allele are given in Methods under “Mice” but we have now also noted this under Flow Cytometry methods (line 542-543).*

In the mRNA sequencing experiment, how many reads were analyzed per sample? Explain this point.

*** *sequencing reads ranged from 50-100 million per sample. We have now included this information in the methods (Lines 558).*
